# A Sensitive Potentiometric Sensor for Isothermal Amplification-Coupled Detection of Nucleic Acids

**DOI:** 10.3390/s18072277

**Published:** 2018-07-14

**Authors:** Kang-Ho Lee, Dongkyu Lee, Jongsu Yoon, Ohwon Kwon, Jaejong Lee

**Affiliations:** 1 Daegu Research Center for Medical Devices, Korea Institute of Machinery and Materials, Daegu 42994, Korea; kangholee6@kimm.re.kr (K.-H.L.); jsyoon@kimm.re.kr (J.Y.); owkwon@kimm.re.kr (O.K.); 2 Nano-Mechanical Systems, Korea Institute of Machinery and Materials, Daejeon 34103, Korea

**Keywords:** potentiometric sensor, surface potential, pH-sensitive sensor, full electrical readout, isothermal amplification, pathogenic bacteria, label-free

## Abstract

A disposable potentiometric sensor was newly developed for the amplification-coupled detection of nucleic acids. The hydrogen-ion is generally released during isothermal amplification of nucleic acids. The surface potential on the oxide-functionalized electrode of the extended gate was directly measured using full electrical circuits with the commercial metal-oxide semiconductor field-effect transistors (MOSFETs) and ring oscillator components, which resulted in cost-effective, portable and scalable real-time nucleic acid analysis. The current-starved ring oscillator changes surface potential to its frequency depending on the square of the variation in pH with a high signal-to-noise ratio during isothermal amplification. The device achieves a conversion rate of 20.5 kHz/mV and a detection resolution of 200 µV for the surface potential. It is demonstrated that the sensor successfully monitors in real-time isothermal amplification of the extracted nucleic acids from *Salmonella* pathogenic bacteria. The in situ variations in the frequency of the pH-sensitive sensor were compared with the results of both a conventional optical device and pH-meter during isothermal amplification.

## 1. Introduction

The analysis of nucleic acids is fundamentally performed in various fields of molecular diagnostics and genomics studies for point-of-care tests (POCT) [1,2]. Amplification-coupled detection has been known to be a standard method to analyze small amounts of nucleic acids. Amplification and detection of nucleic acids are mostly done by polymerase chain reaction (PCR), which measures the fluorescent intensity variation under a precise control thermal cycling [2,3]. However, the fluorescent-based PCR system still requires delicate optical components, which are expensive and relatively bulky for a portable device with costly and time-consuming processes for binding or tagging fluorescent dyes. 

Several attempts have been made to detect and quantify nucleic acids through an electrochemical method for miniaturization and simplification as an alternative to a fluorescent-based optical sensing system [4,5,6,7]. These have exploited variations of electrical signals by reacting a mixture of electrochemical reagents such as intercalating molecules, electroactive reagents, redox probes and ions. However, the detection methods have been limited by the lesser binding efficiency, non-uniform binding and disturbance of additional electrochemical reagents during amplification. In addition, adding reagents usually increases the number of detection steps and preparation time. Another strategy is direct measurement of surface potential variations during amplification [8,9,10,11]. These have measured hydrogen ions released from the sugar-phosphate backbone during the amplification of nucleotide incorporation. The pH-mediated detections of amplification have been demonstrated by portable pH meters [8,9,10]. However, it is quite challenging to improve detection sensitivity within approximately 0.5 pH variations after amplification and to modify device configuration with the temperature control system [9,10].

Ion-sensitive field-effect transistors (ISFETs) have been introduced as an alternative to the glass electrode for pH measurement [11,12,13,14]. Basically, field-effect devices are attractive due to a rapid and robust response, portability, miniaturization and compatibility with commercial microfabrication technology. ISFETs detect net surface charges that are accumulated on the top electrode through shifts of the threshold voltage or change in the drain current [15,16,17]. However, they require post-fabrication to expose the gate oxide layer to the analyte solution. In addition, they are not amenable to the formation of a uniform oxide thickness in a large area and the integration of a reference electrode (Ag/AgCl) in standard complementary metal-oxide-semiconductor (CMOS) processes, which is basically related to signal reproducibility [18]. To overcome those problems, the large ion-sensitive region of the electrodes can be connected with the extended gate electrode of readout metal-oxide semiconductor field-effect transistors (MOSFETs), which is simple and cost-effective without post-fabrication of the MOSFET [19]. Additionally, the stable and reproducible standard electrodes (Ag/AgCl) can be easily printed on a disposable plate because ion-sensitive electrodes are separated from readout circuits.

Here, we have developed the potentiometric sensor which has isolated disposable ion-sensitive electrodes with readout circuits. The readout circuits including a current-starved ring oscillator and commercial MOSFETs are capable of converting variations of frequency from voltage, which means digitalizing surface charges accumulated on the ion-sensitive layer. The readout circuit can amplify electrically the very slight variation in the surface potential during amplification-coupled detection of nucleic acids. The device detects pH changes from frequency variations during the loop-mediated isothermal amplification (LAMP) procedure of nucleic acid. The isothermal temperature was controlled by a programmable resister heater. We have demonstrated real-time amplification of the nucleic acid extracted from *Salmonella* pathogenic bacteria using an electrochemical sensor under the heater. 

## 2. Materials and Methods

### 2.1. Device Structure and First-Order Model of the Device

**Figure 1:**
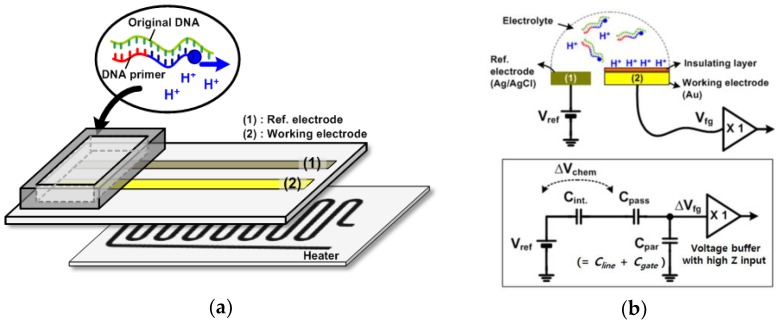
(**a**) Conceptual diagram of an electrochemical chip to amplify nucleic acids; (**b**) electrical models simplified with a first-order model.

Figure 1a shows a conceptual diagram of an electrochemical chip to amplify nucleic acids. The resistive heater is located below the electrodes through an adhesive thermal tape. The disposable chip having working and reference electrodes can be detached from the readout circuits. The electrodes are exposed to the analyte solution in the chamber. The hydrogen ions are released as the polymerization reaction of the nucleic acids proceeds, which was demonstrated in previous reports [11]. The released protons (H^+^) are accumulated on the surface of the electrode, which caused a change of surface potential. Therefore, we can detect the amplification of nucleic acids by electrically monitoring pH variations.

Figure 1b shows the electrical models simplified with a first-order model. As shown in Figure 1b, it is noted that readout circuits detect the chemical signal (H^+^) through capacitive coupling with a Ag/AgCl reference [15]. A reference voltage (*V_ref_*) is applied on the reference electrode. Charged ions (*Q_s_*) at the surface result in the voltage drop across the electrolyte and insulator layers. This voltage is described as a pH-dependent chemical potential (*V_chem_*) between a reference electrode and an insulator’s surface. In addition, the interface between the electrolyte and insulator layer can be modeled with capacitance *C_int_* due to the electrical double layer. The working electrode is connected to the voltage buffer circuit, which has the input characteristic of high impedance: no charges flow through any path in the steady state. The input of the voltage buffer circuit is modeled equivalently with the parasitic capacitance *C_par_*, which includes the gate capacitance *C_gate_* of the MOS transistor and the metal line capacitance *C_line_*. The reference voltage, *V_ref_*, has its ranges set to the operating point of the voltage buffer circuit through the electrolyte solution. Here, the floating gate voltage (*V_fg_*) is capacitively coupled to the sum of *V_ref_* and *V_chem_* via passivation capacitance (*C_pass_*) and scaled down by the *C_par_* [20].

The relationship between the surface charge *Q_s_* and the voltage at the high impedance node, *V_fg_*, is as follows:

(1) $$\Delta V_{\mathrm{fg}}=\frac{C_{\mathrm{pass}}}{C_{\mathrm{pass}}+C_{\mathrm{par}}}\left(V_{\mathrm{ref}}+\frac{\Delta Q_{s}}{C_{\mathrm{int}}}\right)$$

where *ΔV_fg_* is the floating-gate voltage variation responsive to accumulated ions as the pH changes. The *ΔV_chem_* can also be expressed with a change of surface charge *ΔQ_s_* divided by the interface capacitance *C_int_*. Here, it is important to note that *C_par_* has to be much smaller than *C_pass_* for *ΔV_chem_* to be completely transferred to readout circuits. As a result, the electrical charge (*Q_s_*) accumulated on the surface of the electrodes modulates the voltage variation (*ΔV_fg_*), which is the desired signal, at the high impedance node of the voltage buffer circuit. This voltage variation is digitalized by the following ring oscillator circuit. 

### 2.2. Design of an FET Sensor with a Ring Oscillator Circuit

**Figure 2:**
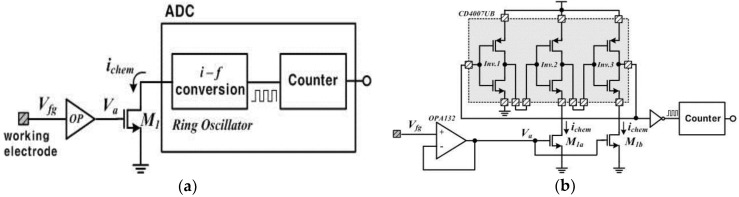
(**a**) Simplified description of the readout circuits; (**b**) detailed description of the readout circuits.

The usage of commercially-proven MOSFETs reduces the time and expense of device manufacture, which results in a low-cost device. Figure 2a shows a simplified description of the readout circuits. The readout circuits are composed of a voltage buffer circuit (*OP*), a current-driving transistor (*M*_1_) and an analog-to-digital convertor (ADC). The voltage buffer circuit is implemented with unity gain using an operational amplifier, OPA132 (Texas Instruments, Sherman, TX, USA), which has the input characteristic of high impedance due to the isolated gates. Thus, it ideally transfers charges induced in the working electrode to the succeeding circuits without any leaky charges. The buffer voltage (*V_a_*) drives the drain current (*i_chem_*) relative to the square of it, when *M_1_* operates in the saturation mode. This is a simple amplifying method through which the device increases the signal-to-noise ratio (SNR). The driven current *i_chem_* is transferred to the ring oscillator and then finally digitalized by a counter, which is called a current-starved oscillator with inherent ADC functionality. Here, the ring oscillator is implemented with an odd number of inverters in a chain. We completed the ring oscillator with a commercial component, CD4007UB (Texas Instruments, USA), connecting three identical inverters in cascade as shown in Figure 2b. The output node of the last inverter is fed back to the input node of the first inverter. These inverters form a closed loop with positive feedback. The circuit oscillates at the specific frequency because this is inherently unstable. Especially in the device, the current *i_chem_* affects the oscillating frequency output because the drain nodes of *M_1a_* and *M_1b_* are connected to the source nodes of the low-side inverters as shown in Figure 2b. In other words, the *i_chem_* controls the time delay of the cascade inverters in the ring oscillator, changing the oscillating frequency. We can derive an equation for the frequency of oscillation if we assume that all inverting stages are identical and each stage provides a delay of *t_d_*. The oscillation frequency *f_o_* of the ring oscillator is given by:

(2) $$f_{o}=\frac{1}{\sum (t_{dHL}+t_{dLH})}$$

where *t_dHL_* and *t_dLH_* are the average propagation delay time at each stage due to an alternative operation of high-to-low and low-to-high.

In Equation (2), the sum of the high-to-low and low-to-high delays is used to calculate the period of the oscillation because each inverter switches twice during a single oscillation period.

Equation (2) can be expressed again as follows:

(3) $$f_{o}=\frac{1}{2t_{d1}+t_{d2HL}+t_{d2LH}+t_{d3HL}+t_{d3LH}}$$

where *t_d_*_1_, *t_d_*_2_ and *t_d_*_3_ are the delay time at the first inverting stage, the second inverting stage and the third inverting stage, respectively. 

Here, the delay per stage is defined as the change in output voltage, *V*, divided by the slew rate, *I_avg_*/*C_load_*, resulting in a delay per stage of *C_load_V*/*I_avg_* [21,22]. The load capacitance *C_load_* is the parasitic capacitance formed between back and forth inverting stages. The current *I_avg_* represents the average current flowing at each stage. In case of a high-to-low operation at the second and third stages, the delay time is affected by *I_chem_* instead of *I_avg_*. Therefore, the oscillation frequency is given by [23]:

(4) $$f_{o}=\frac{1}{2C_{\mathrm{load}}V\left(\frac{2}{I_{\mathrm{avg}}}+\frac{1}{I_{\mathrm{chem}}}\right)}$$

According to Equation (4), it can be known that the oscillation frequency of the ring oscillator is changed as the pH changes by the accumulated ions. Finally, the ADC in the device is completed while counting the oscillator’s pulses by a counter. 

### 2.3. Functionalized Electrodes and Electrical Readout Circuits

**Figure 3:**
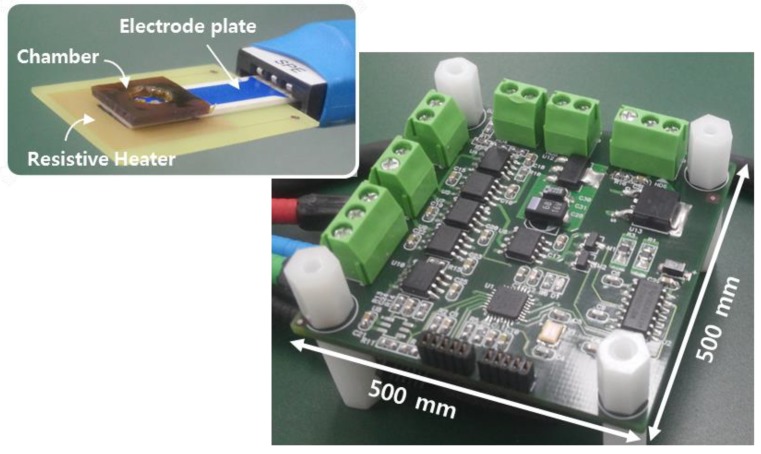
Picture of a prototype circuit platform with functionalized electrodes. The inset shows the photograph of electrochemical sensors on a resistive heater.

Figure 3 shows a prototype platform with functionalized electrodes. We used a commercial screen-printed electrode (BVT Technologies, Brno-Medlánky, Czech Republic), which patterned metal lines on the ceramic substrate. The electrodes have a working electrode of Au and a reference electrode of Ag/AgCl. We deposited a 200-nm-thick layer of oxide on Au for ion-sensitive layer. This sensing layer, being an insulator with a high dielectric constant, accumulates protons at its surface. The electrodes plate has a size of 25.4(L) × 7.26(W) × 0.63(H) mm. The resistive heater is located under the electrodes for an isothermal heating condition during amplification of nucleic acid. The heater generates a resistive heat using a pulse-width modulation (PWM)-based technique. The device enables closed-loop temperature control including a temperature sensor with the resistive heater. Isothermal amplification in this work has the relative simplicity of temperature control compared to that in the thermal cycling application. The chamber, which holds an analyte solution, was covered by taping to prevent solution evaporation during amplification. 

### 2.4. Extraction and Amplification of Nucleic Acids

Cultured *Salmonella* was purchased from DxGene (Seoul, Korea). DNA extraction was conducted using reagents from a tissue mini-preparation kit (Cosmo Genetech, Seoul, Korea), and the extracted DNA was separated by magnetic beads (Bioneer, Daejeon, Korea) following the manufacturer’s protocols. The extraction protocol was described elsewhere [24]. The initial concentration of the extracted DNA following the protocol was measured to be approximately 37 ng/µL (at 10^9^ CFU/mL *Salmonella*) using a spectrophotometer (Bibby Scientific, Staffordshire, U.K.). The buffer condition is a very important factor for the detection of pH variation in the isothermal amplification, as mentioned in previous papers [8,9,10,11]. Buffer was made by mixing of 50 mM KCl, 5 mM MgSO_4_, 1 M betaine, 5 mM NH_4_Cl and 0.1% Triton X-100 in D.I. water. The pH of the buffer was set to 8.5. For isothermal amplification, dNTP, primer set (I and II) and bst polymerase were purchased from Takara (Shiga, Japan)), Bioneer (Daejeon, Korea) and Enzynomics (Daejeon, Korea), respectively. Extracted DNA solution was mixed with buffer solution, dNTP, primer set, bst polymerase and D.I. water. Before amplification-coupled detection using FET, the buffer condition was confirmed by a commercial PCR device (Applied Biosystems, Carlsbad, CA, USA) and by gel electrophoresis, as shown in Supplementary Materials (Figure S1). Considering heat loss to the open surface, isothermal amplification was conducted at 61 °C (commercial PCR device) and 65 °C (prototype devices) for 60 min. The profiles of increasing temperatures from room temperature to 65 °C using a resistive heater were very reproducible, as shown in the Supplementary Materials (Figure S2). Isothermal amplification kits were also purchased from DxGene (Seoul, Korea). Agarose was purchased from Bio-Rad (Seoul, Korea).

## 3. Results

### 3.1. Performance of the Designed Sensor

**Figure 4:**
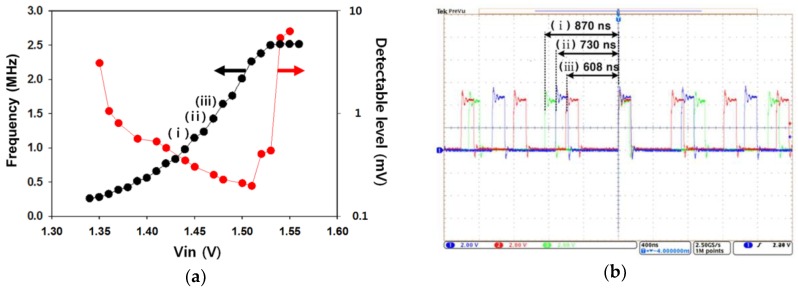
(**a**) Oscillation frequency of the ring oscillator and detection resolution for the surface potential as the input voltage changes; (**b**) operating waveforms of the ring oscillator according to the three different conditions for the input voltage.

In Figure 4, the electrical performance of the prototype is verified. Figure 4a shows the oscillation frequency of the ring oscillator and the detection resolution for the surface potential as the input voltage (*V_in_*) changes. The input voltage, *V_in_*, represents the surface potential coupled to the reference voltage. Here, the outputs of the ring oscillator responsive to the input voltage were investigated to find the best operating range for the significant results in this work. We observed that the device had a suitable range for the input voltage of 1.38 V to 1.53 V and was capable of detecting a surface potential of as little as 200 µV. The previous ISFET devices were normally limited by their minimum detectable level (several mV) of the surface charge, because small fluctuating signals are buried by the noise components of the FET [25,26]. Our device achieved a significant performance of detection resolution compared to that of the ISFET device. Here, the maximum frequency conversion rate was a 20.5 kHz/mV. Figure 4b shows the operating waveforms of the ring oscillator according to the three different conditions for the input voltage. We expected that the surface potential would increase as the chemical ions (H^+^) were accumulated on the surface of the working electrode, and the oscillation frequency of the ring oscillator increased.

### 3.2. Isothermal Amplification of Nucleic Acids

**Figure 5:**
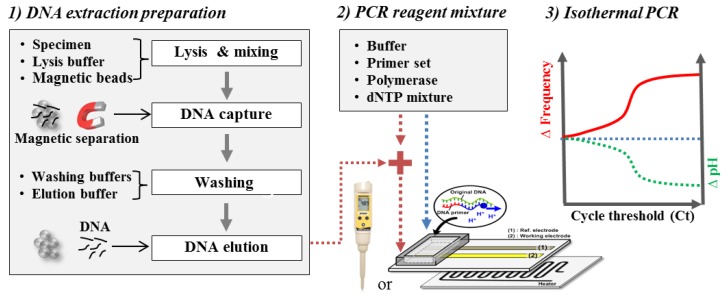
Schematic diagram of the experimental procedures.

The extracted nucleic acids solution was mixed with PCR reagent mixture after extracting nucleic acids from *Salmonella* pathogenic bacteria following the manufacturer’s protocols. The isothermal amplifications of the mixture solution with/without the extracted nucleic acids were conducted. The frequency variation in the potentiometric sensor and the pH variation in the portable pH meter were monitored during isothermal amplification, as shown in Figure 5.

**Figure 6:**
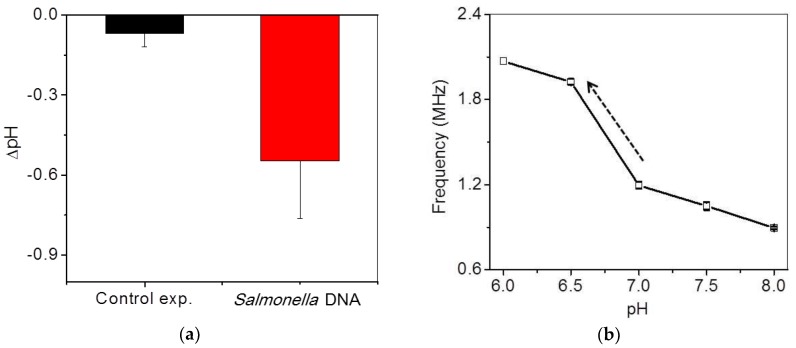
(**a**) Ex-situ pH variations of DNA and control samples using a commercial pH detector after isothermal amplification; (**b**) frequency variations with respect to standard pH.

Detection of *Salmonella* DNA using isothermal amplification was also confirmed by a commercial pH detector (Eutech, Singapore) in order to realize how much pH varies during isothermal amplification in this system, as shown in Figure 6a. One hundred microliters of the final test solution were prepared in a small chamber immersed in a water bath at 65 °C. The variation in the pH of the extracted DNA sample was measured to be approximately −0.6 ± 0.2 during isothermal amplification for 60 min, whereas that of a control sample without DNA was measured to be approximately −0.07 ± 0.05 at the same conditions. The slight pH variation of the control sample was attributed to the temperature change. However, measurement using a commercial pH detector was not quite convenient due to the large pH probe, uncomplete sealing and large equipment. 

To optimize the system condition in the specific region where pH variation occurs during isothermal amplification, frequency shifts were calibrated to pH variation with standard solutions. The initial pH of the mixing DNA solution was approximately in the range of 7 to 7.5. Since the frequency variation with respect to pH change does not show a linear relationship, the highest frequency variation should be optimized in the range of pH 7 to 6.5 in this system, as shown in Figure 6b. The slope of the region was fitted to be 730 kH/0.5 pH. The frequency signal was saturated at over 2 MHz. 

**Figure 7:**
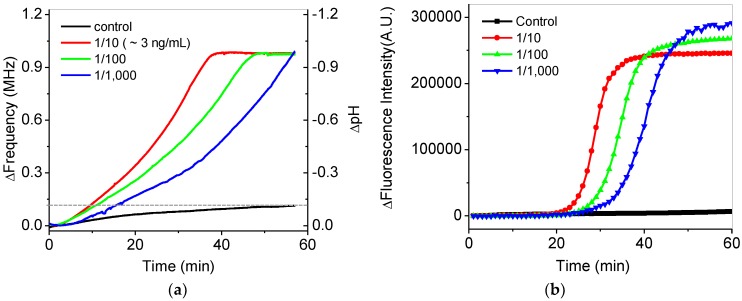
(**a**) In situ frequency and pH variations of serially-diluted samples during isothermal amplification. The dotted line shows the final frequency change of the control experiment. (**b**) Variations in the fluorescence intensity of the serially-diluted samples during the isothermal amplifications using a commercial PCR device.

Figure 7a shows the in situ frequency and pH variations during isothermal DNA amplification at 65 °C for 60 min. Real-time variations of frequency for the serially-diluted DNA samples from 10 to 1000 times in the extracted DNA samples were tested for quantification. Faster increases of the frequency were shown as higher concentrations of the extracted DNA samples were included in the final mixing solution. The final frequency variations of the DNA samples were saturated to be 1 MHz, which reached the maximum countable values as pH was changed from around 7.5 to 6.5. For the control experiment, we confirmed that the variations in the frequency and pH of a control sample without DNA were negligible during isothermal amplification, as shown by the black line in Figure 7a. The frequency variation for the control sample was measured to be 140 kHz after amplification, which is seven-times smaller than that for DNA samples. The variations of a 1-MHz frequency were roughly calibrated to −1 pH variation using Figure 6b. The variations of pH in this system were very similar to the previous results [8,9]. To compare these results with those of a commercial PCR device, fluorescence-based detection for the isothermal amplifications of the same DNA samples was obtained by using the commercial PCR device, as shown in Figure 7b. Similar results between Figure 7a,b were observed except showing zero intensity below a cutoff value at the initial stage in Figure 7b due to data processing of the commercial device. 

**Figure 8:**
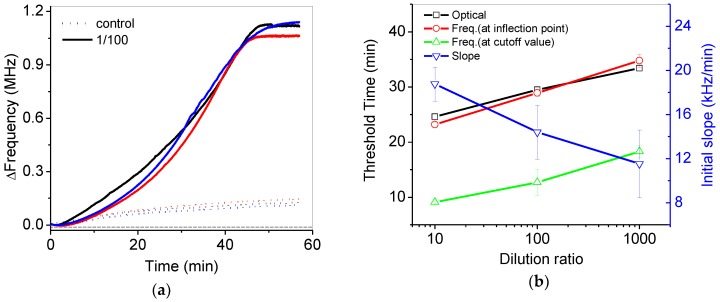
(**a**) Reproducible frequency variations in the 1/100 diluted and control samples during isothermal amplification; (**b**) the estimated threshold time from variations in the fluorescence intensity (black open square) and frequency by using various methods such as the frequency at the inflection point (red open square), the frequency at the cutoff value of the control experiment (green open triangle) and the initial slope at early stage (blue reverse open triangle).

Figure 8a shows reproducible frequency variations in the 1/100 diluted and control samples during isothermal amplification. Figure 8b shows the threshold time (Ct) at the inflection point from the data of Figure 7a,b. Basically, the threshold time (black open square) can be calculated by the point of intersection between best-fitted lines at the early and later stage of variations in fluorescence intensity from the commercial PCR device. In a similar way, the threshold time (red open circle) was obtained from as for the frequency variations. The slopes at early stage of frequency variations in Figure 7a were estimated to be 18.7 to 11.5 kHz/min for 10 to 1000 diluted samples, respectively, but slopes at the later stage of frequency variations were relatively larger. The threshold time from pH variation (red open circle) was quite similar to the threshold time from the results of the commercial PCR device (black open square). The initial slope at the early stage was gradually reduced with respect to the increase of the dilution ratio. Analysis of the initial slope variations (blue reverse open triangle) in the frequency can also be a good alternative for quantitative analysis. If the control result were considered as a cutoff value at the initial stage, the threshold time from pH variations (green open triangle) could be estimated to be around 10 min, as shown in Figure 8.

The performance of PCR detection using the potentiometric sensor was comparable to that using the commercialized PCR device. The analysis time of both systems was almost the same due to the requirement of isothermal amplification time. The Ct values from the potentiometric sensor were similar to those from the commercialized optical detector, as shown in Figure 8b. It can be further improved with the optimization of the experimental conditions and system packaging. However, the potentiometric sensor can be over 10-times less expensive and smaller than the optical modules of the fluorescence detector. In addition, the cost of a single test with fluorescence-modified DNA in PCR reagents was usually two- or three-times more expensive than that without fluorophores in PCR reagents. Therefore, the potentiometric sensor can be utilized for an inexpensive and portable PCR electrical detector having comparable performance to the commercialized optical detector. 

## 4. Conclusions

In this study, a disposable potentiometric sensor was developed for the detection of the pH variations during the LAMP procedure of nucleic acids. The pH-sensitive sensor directly measures the surface potential on the oxide-functionalized electrode using FET-based readout circuits. The readout circuits include a ring oscillator in order to amplify signals electrically in the specific region and to digitalize surface charges’ accumulation on the ion-sensitive layer. The ring oscillator circuit with commercial MOSFETs is cost-effective and stable for mass production. It was demonstrated that the sensor successfully monitors in real time the isothermal amplifications of the extracted DNA from *Salmonella* pathogenic bacteria. The in situ profiles in the changes of frequency and pH were compared with the fluorescent intensity signals of the commercial PCR device. Therefore, we developed a small sensor system for the detection of LAMP, which can be potentially applied for potable miniaturized PCR devices in the area of point-of-care (POC) tests, sensitive virus detection and foodborne bacterial detection.

## Supplementary Materials

The following are available online at http://www.mdpi.com/1424-8220/18/7/2277/s1: Figure S1: Electropherograms of LAMP in the commercial or developed buffer; Figure S2: Temperature profiles after setting the temperature at 65 °C.
