# Supplementary material for: A Sensitive Potentiometric Sensor for Isothermal Amplification-Coupled Detection of Nucleic Acids
Source: Sensors (Basel). 2018 Jul 14;18(7):2277. doi: 10.3390/s18072277 (PMC6068556; doi:10.3390/s18072277)

A Sensitive Potentiometric Sensor for Isothermal Amplification-Coupled Detection of Nucleic Acids

Kang-Ho Lee [1,†], Dongkyu Lee [1,*,†], Jongsu Yoon [1], Ohwon Kwon [1] and Jaejong Lee [2,*]

1 Daegu Research Center for Medical Devices, Korea Institute of Machinery and Materials, Daegu 42994, Korea; kangholee6@kimm.re.kr (K.-H.L.); jsyoon@kimm.re.kr (J.Y.); owkwon@kimm.re.kr (O.K.)
2 Nano-Mechanical Systems, Korea Institute of Machinery and Materials, Daejeon 34103, Korea
* Correspondence: dongkyu@kimm.re.kr (D.L.); jjlee@kimm.re.kr (J.L.)
† These authors contributed equally to this work.

DNA Extraction Protocol

The microbe sample was separated using a centrifuge (at 8000 RPM for 5 min). The 200 μL of tissue lysis buffer and 20 μL of proteinase K (20 mg/mL) were added and reacted with the sample for 5 min in order to obtain a high molecular weight of Salmonella DNA, and then it was mixed with 50 μL of magnetic bead solution (20 mg/mL) and 200 μL of high purified ethanol. The 400 μL of DNA binding buffer on magnetic beads was mixed with the solution. Two times of washing processes with 700 μL of washing buffer I and II were followed to remove the proteins and impurities in the solution. The 400 μL of elution buffer was added to collect the extracted target DNA.

Figure S1. Electropherograms of LAMP in the commercial or developed buffer.

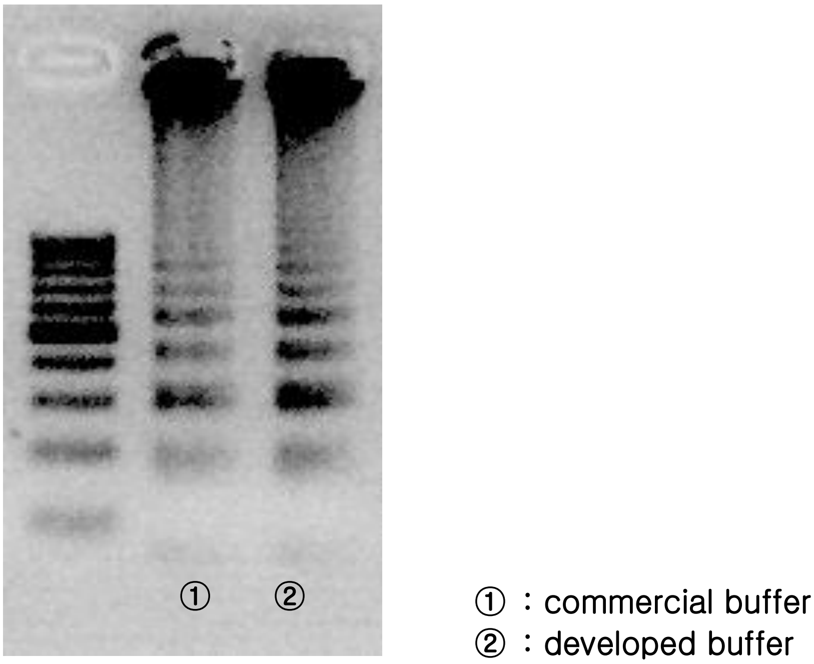

Figure S2. Temperature profiles after setting temperature at 65 °C.

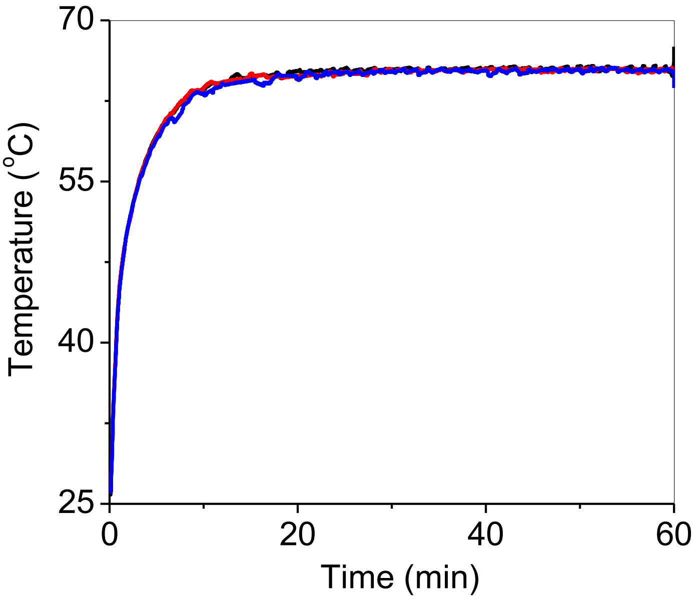

Supplement: Supplementary file 1 [file sensors-18-02277-s001.pdf]
